# Transumbilical single-site double-port laparoscopic appendectomy in children. Report of a single center experience with comparison with three-port laparoscopic appendectomy

**DOI:** 10.3389/fped.2025.1541702

**Published:** 2025-07-18

**Authors:** Hong Qin, Hongzhen Liu, Tian Xia, Bingqiang Tang, Shisong Zhang, Lulu Wang

**Affiliations:** ^1^ Department of General Surgery, Children’s Hospital Affiliated to Shandong University, Jinan, Shandong, China; ^2^ Department of Endocrinology, Jinan Central Hospital Affiliated to Shandong First Medical University, Jinan, China

**Keywords:** minimally invasive surgery, pediatric, laparoscopic appendectomy, transumbilical, surgical treatment

## Abstract

**Objective:**

Transumbilical single-site double-port laparoscopic appendectomy (TSSDPLA) represents an innovative and minimally invasive approach for managing acute appendicitis. This study aims to compare the clinical outcomes of TSSDPLA vs. three-port laparoscopic appendectomy (TPLA) in a pediatric series.

**Method:**

A retrospective analysis was conducted between July 2023 and July 2024 at the Department of General Surgery, Children's Hospital affiliated to Shandong University. The patients were categorized into two groups: the TSSDPLA group and the TPLA group. Outcomes including patient demographics, leukocyte count upon arrival, duration of the operation, costs associated with the operating room, and any complications encountered.

**Results:**

The results indicated no significant differences between the TSSDPLA and TPLA groups in terms of age, gender, leukocyte counts, liquid diet, length of hospital stay, pathology or complications. However, the mean operating room costs were significantly lower in the TSSDPLA group (*p* < 0.001). Additionally, the mean operation time for the TSSDPLA group was marginally longer compared to the TPLA group.

**Conclusions:**

TSSDPLA is a reliable technical technique for the management of acute appendicitis. This approach offers several advantages, including improved cosmetic outcomes, reduced surgical trauma, and lower costs.

## 1 Introduction

Appendicitis is the most common indication of abdominal surgery in children. Laparoscopic appendectomy has emerged as the gold standard for the surgical treatment of appendicitis in children (1–3). However, significant variability exists among centers regarding the operative approaches to pediatric appendicitis. In this context, we present a novel alternative to the three-port laparoscopic approach to evaluate and compare the outcomes of two minimally invasive techniques in pediatric field: TSSDPLA and TPLA procedures.

## 2 Materials and methods

### 2.1 General information

This is a retrospective single-pediatric center matched case–control study to evaluate the safety of the TSSPDLA technique. All pediatric patients who underwent TAADPLAs or TPLA between July 2023 and July 2024 were queried from our institutional database. Exclusion criteria for the study included: (1) children younger than 3 years of age; (2) a preoperative history exceeding 3 days, (3) conversion to open appendectomy via a McBurney incision, and (4) combined with another surgical procedure.

Patients in the TSSDPLA group were matched with TPLA controls based on age, gender, leukocyte counts, liquid diet, length of hospital stay, pathological findings, operative duration,wound infections,complications (including ileus, intestinal occlusion due to adhesions, intra-abdominal abscess formation, and readmissions occurred within the first 30 days postoperatively) and cost associated with each group using the Greedy match method. The Greedy match method matches cases to controls based on their propensity to receive the intervention. It matches each case to the control with the minimal difference in propensity score within a predefined limit or as predetermined differences in specific variables.

All laparoscopic appendectomy procedures were performed by experienced surgeons from the same cohort. Postoperative care included administration of metronidazole and a third-generation cephalosporin to all children. Individual treatment regimens were adjusted based on postoperative clinical symptoms and antimicrobial susceptibility test results. Patients were discharge once dietary intake and inflammatory markers returned to normal levels.

All data collection and screening procedures were conducted in accordance with the principles outlined in the Declaration of Helsinki. Due to the retrospective nature of the study, the medical research center and institutional review board of Children's hospital affiliated to Shandong University waived the need of obtaining informed consent. (SDFE-IRB/T-2024071).

### 2.2 Description of technique

#### 2.2.1 TSSDPLA: transumbilical single-site double-port laparoscopic appendectomy

**Figure 1:**
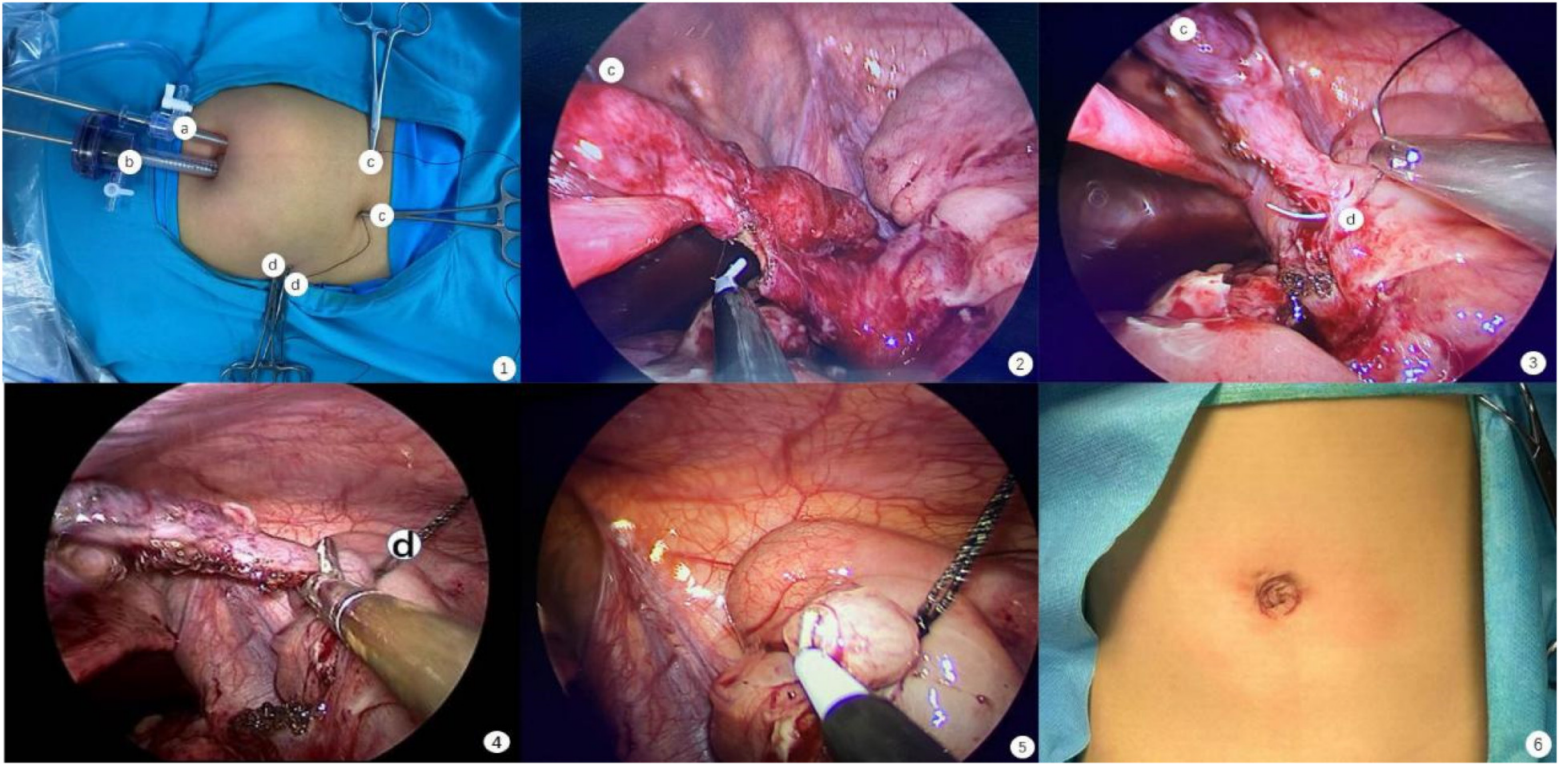
The procedure of TSSDPLA. 1. The position of Trocar and Mersilk; 2. Cauterise the appendicular artery *in situ*; 3. Suture Mersilk through serosa of appendix; 4. Cut the appendix; 5. Appendiceal stump closure; 6. The picture of sutured wound.

A 10-mm trocar was utilized to establish pneumoperitoneum at the umbilical edge in the 10 o'clock position, followed by the insertion of a 5 mm trocar at the 5 o'clock position. (Figure 1(1)). The surgeon positioned themselves on the left side of the pediatric patient, using the left hand to hold the laparoscope, while the right hand manipulated the forceps. After intra-abdominal suspension of the appendix, with the mesoappendix oriented toward the surgeon, the hook was replaced, and the mesoappendix was cauterized to ensure complete hemostasis, skeletonization, and dissection up to the root of the appendix (Figure 1(2)). Another 2-0 Mersilk suture was inserted from the right anterior superior iliac spine, leaving the end of the thread external. The thread was secured to the serosa at the base of the appendix, and the appendix was ligated (Figure 1(3)). The appendix was transected 0.5 cm distal to the ligature and coagulated at the level of the transection (Figures 1(4–5)). Abscess fluid and blood were aspirated from the abdominal cavity for drug sensitivity testing. The Mersilk suture was excised intra-abdominally, the appendix was extracted through the 10-mm trocar, and the paraumbilical incision was closed with sutures. (Figure 1(6)).

#### 2.2.2 TPLA: three-port laparoscopic appendectomy

(1) A 10 mm trocar is inserted in an “open” fashion through an infraumbilical incision. A 5 mm 30° laparoscope was introduced, and under direct visualization, two 5 mm operative trocars were placed in the left flank and suprapubic region.
(2) In cases where the TSSSDLA procedure was challenging, an additional 5 mm trocar was at the suprapubic level.

### 2.3 Statistical analysis

SPSS 25.0 (IBM, Armonk, NY, USA) was used for the statistical calculations. Continuous variables that obeyed the normal distribution were expressed in the form of Mean ± SD. Shapiro–Wilk(S–W) test and Mann–Whitney *U*-test were used for ranked results with and without a standard distribution, respectively, and chi-square test or ANOVA was used for comparison between groups. Values of *p* < 0.05 were considered statistically significant.

## 3 Results

**Figure 2:**
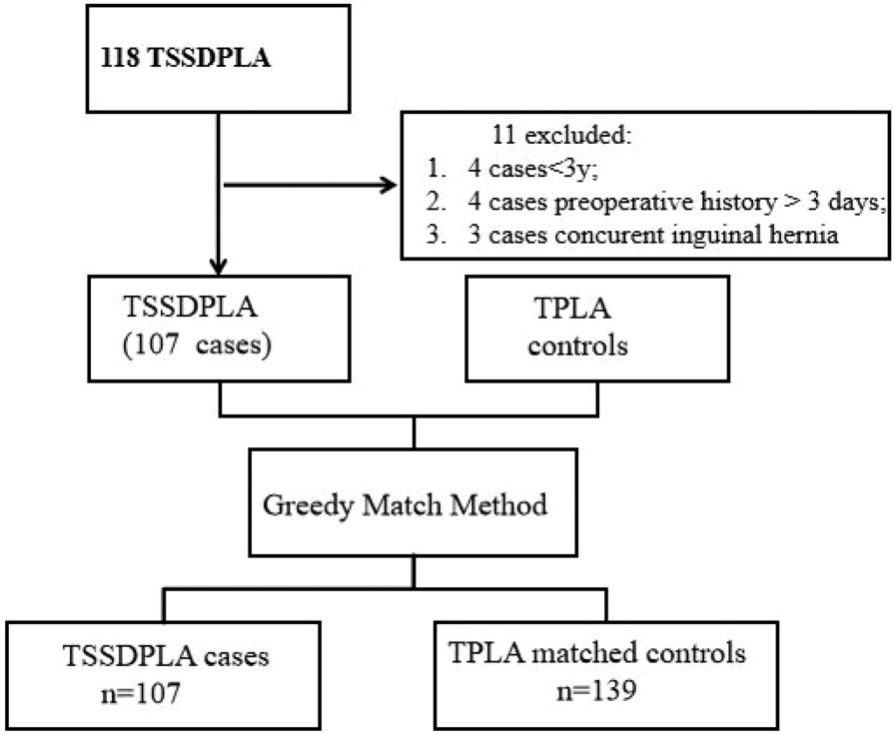
Greedy method case–control matching. TSSDPLA, transumbilical single-site double-port laparoscopic appendectomy. TPLA three-port laparoscopic appendectomy.

TSSDPLA enrollment procedure:A total of 128 patients underwent TSSDPLA at our institution. After applying exclusion criteria (age <3 years, preoperative symptom duration >3 days, or concurrent surgical procedures), the final cohort comprised 107 patients (46 female individuals, 43.0%) (Figure 2).

Case–control matching was performed, including 139 controls (62 female patients, accounting for 44.60%). There were no cases requiring conversion to open appendectomy; however, 12 cases were converted from TSSDPLA to TPLA. The mean age of patients was 6.49 years in the TSSDPLA group and 6.14 years in the TPLA group. The average leukocyte count was 14.19 × 10^9 ^/L in the TSSDPLA group and 14.01 × 10^9 ^/L in the TPLA group. The mean duration of liquid diet was 1.07 days in the TSSDPLA group and 1.14 days in the TPLA group. The average length of hospital stay was 6.50 days in the TSSDPLA group and 7.17 days in the TPLA group. Postoperative pathological diagnoses in the TSSDPLA group included acute pure appendicitis in 11 (18.28%) children, acute suppurative appendicitis in 34 (31.78%), and perforated appendicitis in 62 (57.94%) patients. In the TPLA group, these figures were 13 (9.36%), 45 (32.37%), and 81 (58.27%), respectively. Complications within the first 30 postoperative days, including wound infections, ileus, intestinal occlusion due to adhesions, intraabdominal abscess formation, and readmissions, occurred in 17 patients in the TSSDPLA group and 31 patients in the TPLA group. No significant differences were observed between the groups in terms of gender (*p* = 0.800), age (*p* = 0.249), leukocytes (*p* = 0.700), time for liquid diet (*p* = 0.107), length of stay (*p* = 0.16), pathological classification (*p* = 0.970) and complications (*p* = 0.208).

A statistically significant difference was observed in the cost of surgical materials, with the TPLA group incurring higher expenses (*p* < 0.001). The mean cost of surgical materials was $217.38 ± 2.81 for TSSDPLA and $325.84.07 ± 2.78 for TPLA, it was (Table 1). The costs associated with anesthesia medications and postoperative anti-infection therapy were approximately equivalent due to the comparable average duration of surgery and length of hospital stay. Consequently, the TPLA group experienced higher overall hospital expenses.

**Table 1:** Demographic, clinical, outcome and costs data (TSSDPLA vs. TPLA).

Items		Two-port groups	Three-port groups	*χ*^2^/F	*P* **
Gender	Male	61 (57.00%)	77 (55.40%)	0.064	0.800
	Female	46 (43.00%)	62 (44.60%)		
Wound infections	Yes	2 (1.87%)	3 (2.16%)	0.101	0.874
	No	105 (98.13%)	136 (97.84%)		
Complication*	Yes	15 (14.02%)	28 (20.14%)	6.550	0.211
	No	92 (85.98%)	111 (79.86%)		
Pathology	Pure	11 (18.28%)	13 (9.36%)	0.030	0.970
	Suppurative	34 (31.78%)	45 (32.37%)		
	Perforated	62 (57.94%)	81 (58.27%)		
Age (years)	6.49 ± 2.66	6.14 ± 2.16	7.940	0.249	
Leukocytes (10^9 ^/L)	14.19 ± 4.21	14.01 ± 4.06	0.385	0.700	
Liquid diet (days)	1.07 ± 0.29	1.14 ± 0.37	9.759	0.107	
Length of stay (days)	6.50 ± 1.66	7.17 ± 2.59	23.316	0.16	
Operation time (min)	35.18 ± 8.46	30.06 ± 13.74	6.446	0.001	
Operating room costs ($)	217.38 ± 2.81	325.84.07 ± 2.78	0.026	0.001	

* Ileus, intestinal occlusion by adhesions, intraabdominal abscess formation, readmissions, etc occurred within the first 30 days after surgery.

** Statistically significant where *P* < 0.05.

## 4 Discussion

AS summarized in a 2018 Cochrane review, Jaschinski et al. reported the advantages of LA over OA and LA, establishing LA as the current standard surgical procedure for treating acute appendicitis (4). Advancements from traditional three-port laparoscopy to techniques such as laparoscopy assisted by small incisions, transumbilical laparoscopic-assisted appendectomy, and further to transumbilical single-site three-channel laparoscopy, have focused on minimizing scarring and enhancing aesthetic outcomes (5–7). In this study, we present the surgical techniques involved in TSSDPLA implied that the technique is safe and feasible.

Although TPLA is considered the most traditional and straightforward approach, it requires two incisions in the lower abdominal wall, which may result in a risk of injury to the inferior epigastric artery or the iliohypogastric nerve due to trocar placement (8, 9). In contrast, both small-incision assisted laparoscopy and transumbilical single-incision laparoscopy typically require longer incisions, measuring over 1.5–2.0 cm (6). Additionally, extracorporeal appendectomy may increase the risk of incision infection due to direct contact between the appendix and the umbilical incision (7). Transumbilical three-channel laparoscopy represents an advancement over conventional three-port laparoscopy. However, the confined positioning at the umbilicus exacerbates the ‘chopstick effect’ of the laparoscope and instruments, significantly increasing procedural complexity (10). This technique utilizes customized port such as telescope incorporated with a working channe, a multichannel port, or a glove port. In contrast to single-site laparoscopy assisted by a double-trocar system, transumbilical single-site double-port laparoscopic appendectomy circumvents the need for an additional 2 mm incision in the right lower abdomen (11).

We introduced several innovations in TSSDPLA, such as positioning the umbilical incisions at the upper right and lower left of the umbilicus, rather than in the same plane, to maximize the distance between the two trocars. Furthermore, the trocars were inserted at varying depths to minimize the risk of collision and interference both intro- and extro-abdominally cavity. The surgeon independently performs laparoscopy and intraperitoneal manipulation, improving coordination between the visual field and the operative surface. Through the aforementioned procedures, we have endeavored to minimize the interference caused by the chopstick effect.

Due to the lack of triangulation and the rigid relationship between the angle of visualization and the instruments, we opted to introduce 2-0 Mersilk suture from the suprapubic region into the abdominal cavity instead of a new trocar. This maintains the vertical orientation of the appendix, ensures complete exposure of both the appendix and the mesoappendix, thereby minimizing the risk of collateral injury (12).

We refined the technique for appendiceal stump closure (ASC) by reintroduces a 2-0 Mersilk suture above the anterior superior iliac spine. The suture is passed through the serosal layer at the base of the appendix and then wrapped around. ASC was accomplished through coagulation and the application of a Mersilk coil (13, 14), as illustrated in Figures 1(3–5).

The study found that TPLA incurred significantly higher costs than TSSDPLA, primarily due to the increased number of trocars used. This elevated cost of TPLA is not offset by a reduction in hospital stay duration, as hospitalization durations were similar between the two groups. Notably, TSSDPLA via the umbilicus offers several advantages for treating acute appendicitis in children. This technique reduces the size of the abdominal wall puncture, minimizing abdominal wall injury and postoperative pain while preserving the umbilicus's natural appearance for improved aesthetic outcomes. Thus, in addition to clinical benefit, TSSDPLA is associated with reduced overall costs due to the use of fewer trocars and no customized devices (15).

We are aware of the limitations of TSSDPLA such as the difficult mobilization of the adherent appendix, and the dissection of sub-serous or retro-cecal appendices. Additionally, this study has inherent limitations due to its retrospective design. Future studies of this technique should across all age groups and incorporate subjective assessments of pain and cosmesis.

## 5 Conclusions

In conclusion, the TSSDPLA approach for managing appendicitis in pediatric patients retains benefits of TPLA. By incorporating external traction using Mersilk, this technique reduces the need for specialized instruments, minimizes the use of trocars, and consequently decreases the consumption of disposable materials, thereby lowering surgical costs. Additionally, it enhances postoperative cosmetic outcomes by eliminating visible scarring. Therefore, TSSDPLA represents a valuable and safe advancement over TPLA. Given the high prevalence of appendicitis, even minor cost improvements per case can lead to significant savings in healthcare resources and societal costs.

## Data Availability

The datasets presented in this study can be found in online repositories. The names of the repository/repositories and accession number(s) can be found below: doi: 10.17632/dx9sbkfg3r.2.

## References

[1] Snyder MJ Guthrie M Cagle S. Acute appendicitis: efficient diagnosis and management. Am Fam Physician. (2018) 98(1):25–33. 30215950

[2] Moris D Paulson EK Pappas TN. Diagnosis and management of acute appendicitis in adults: a review. JAMA. (2021) 326(22):2299–311. 10.1001/jama.2021.20502 34905026

[3] Krzyzak M Mulrooney SM. Acute appendicitis review: background, epidemiology, diagnosis, and treatment. Cureus. (2020) 12(6):e8562. 10.7759/cureus.8562 32670699 PMC7358958

[4] Jaschinski T Mosch CG Eikermann M Neugebauer EA Sauerland S. Laparoscopic versus open surgery for suspected appendicitis. Cochrane Database Syst Rev. (2018) 11(11):CD001546. 10.1002/14651858.CD001546.pub4 30484855 PMC6517145

[5] Bindi E Nino F Pierangeli F Ilari M Bollettini T Chiarella E Transumbilical laparoscopic-assisted appendectomy versus laparoscopic appendectomy in children: a single center experience. Pediatr Med Chir. (2023) 45(1):306. 10.4081/pmc.2023.306 37114377

[6] Di Saverio S Podda M De Simone B Ceresoli M Augustin G Gori A Diagnosis and treatment of acute appendicitis: 2020 update of the WSES Jerusalem guidelines. World J Emerg Surg. (2020) 15(1):27. 10.1186/s13017-020-00306-3 32295644 PMC7386163

[7] Borges-Dias M Carmo L Lamas-Pinheiro R Henriques-Coelho T Estevão-Costa J. Trans-umbilical laparoscopic-assisted appendectomy in the pediatric population: comparing single-incision and 2-trocar techniques. Minim Invasive Ther Allied Technol. (2018) 27(3):160–3. 10.1080/13645706.2017.1399279 29130739

[8] Faulkner J Beeson S Fox S Yon J Hope W. Inferior epigastric artery pseudoaneurysm following laparoscopic appendectomy. Am Surg. (2023) 89(9):31348231161682. 10.1177/00031348231161682 37344963

[9] Quevedo Orrego E Robla Costales J Rodríguez Aceves C Diana Martín R González Álvarez A Socolovsky M. Neuropathic inguinal pain due to nerve injury after a laparoscopic appendectomy: first pediatric case described in the literature. Childs Nerv Syst. (2021) 37(6):1825–30. 10.1007/s00381-021-05177-w 33904935

[10] Joseph RA Goh AC Cuevas SP Donovan MA Kauffman MG Salas NA “Chopstick” surgery: a novel technique improves surgeon performance and eliminates arm collision in robotic single-incision laparoscopic surgery. Surg Endosc. (2010) 24(6):1331–5. 10.1007/s00464-009-0769-8 20033723

[11] Geng J Yuan J Kong X Wu M Zeng L Hu Y Laparoscopic traction device for assistance of 2-port laparoscopic appendectomy. Am Surg. (2021) 87(9):1511–3. 10.1177/0003134820920221 33497250

[12] Linnaus ME Ostlie DJ. Complications in common general pediatric surgery procedures. Semin Pediatr Surg. (2016) 25(6):404–11. 10.1053/j.sempedsurg.2016.10.002 27989365

[13] Erdoğan A Türkan A. Comparison of handmade endoloop versus polymeric endoclip for stump closure in laparoscopic appendectomy. Cureus. (2021) 13(7):e16302. 10.7759/cureus.16302 34381657 PMC8352043

[14] Antoniou SA Mavridis D Hajibandeh S Hajibandeh S Antoniou A Gorter 7R Optimal stump management in laparoscopic appendectomy: a network meta-analysis by the minimally invasive surgery synthesis of interventions and outcomes network. Surgery. (2017) 162(5):994–1005. 10.1016/j.surg.2017.07.013 28864100

[15] Malhotra L Pontarelli EM Grinberg GG Isaacs RS Morris JP Yenumula PR. Cost analysis of laparoscopic appendectomy in a large integrated healthcare system [published correction appears in surg endosc. 2021;36(1):800–807]. Surg Endosc. (2022) 36(1):800–7. 10.1007/s00464-020-08266-0 33502616

